# CD137L Inhibition Ameliorates Hippocampal Neuroinflammation and Behavioral Deficits in a Mouse Model of Sepsis-Associated Encephalopathy

**DOI:** 10.1007/s12017-023-08764-z

**Published:** 2023-10-05

**Authors:** Fang Qiu, Yueming Liu, Yang Liu, Zhuyun Zhao, Lile Zhou, Pengfei Chen, Yunbo Du, Yanmei Wang, Huimin Sun, Changchun Zeng, Xiaokang Wang, Yuqiang Liu, Haobo Pan, Changneng Ke

**Affiliations:** 1 Department of Burn and Plastic Surgery, Shenzhen Longhua District Central Hospital, Shenzhen, 518110 Guangdong China; 2 Center for Human Tissues and Organs Degeneration, Shenzhen Institutes of Advanced Technology, Chinese Academy of Sciences, Shenzhen, 518055 Guangdong China; 3 Guangdong Medical University, Zhanjiang, Guangdong China; 4 Department of Traumatic Orthopedics, Shenzhen Longhua District Central Hospital, Shenzhen, China; 5 Department of Critical Care Medicine, Shenzhen Longhua District Central Hospital, Shenzhen, Guangdong China; 6 Department of Medical Laboratory, Shenzhen Longhua District Central Hospital, Shenzhen, Guangdong China; 7 Department of Pharmacy, Shenzhen Longhua District Central Hospital, Shenzhen, Guangdong China; 8 Department of Anesthesiology, Shenzhen Second People’s Hospital, The First Affiliated Hospital of Shenzhen University, Shenzhen, 518025 Guangdong China

**Keywords:** CD137L, Microglia, Inflammation, Anxiety, Cognition, TKS-1

## Abstract

Anxiety manifestations and cognitive dysfunction are common sequelae in patients with sepsis-associated encephalopathy (SAE). Microglia-mediated inflammatory signaling is involved in anxiety, depression, and cognitive dysfunction during acute infection with bacterial lipopolysaccharide (LPS). However, the molecular mechanisms underlying microglia activation and behavioral and cognitive deficits in sepsis have not been in fully elucidated. Based on previous research, we speculated that the CD137 receptor/ligand system modulates microglia function during sepsis to mediate classical neurological SAE symptoms. A murine model of SAE was established by injecting male C57BL/6 mice with LPS, and cultured mouse BV2 microglia were used for in vitro assays. RT-qPCR, immunofluorescence staining, flow cytometry, and ELISA were used to assess microglial activation and the expression of CD137L and inflammation-related cytokines in the mouse hippocampus and in cultured BV2 cells. In addition, behavioral tests were conducted in assess cognitive performance and behavioral distress. Immunofluorescence and RT-qPCR analyses showed that hippocampal expression of CD137L was upregulated in activated microglia following LPS treatment. Pre-treatment with the CD137L neutralizing antibody TKS-1 significantly reduced CD137L levels, attenuated the expression of M1 polarization markers in microglia, and inhibited the production of TNF-α, IL-1β, and IL-6 in both LPS-treated mice and BV2 cells. Conversely, stimulation of CD137L signaling by recombinant CD137-Fc fusion protein activated the synthesis and release of pro-inflammatory cytokines in cultures BV2 microglia. Importantly, open field, elevated plus maze, and Y-maze spontaneous alternation test results indicated that TKS-1 administration alleviated anxiety-like behavior and spatial memory decline in mice with LPS-induced SAE. These findings suggest that CD137L upregulation in activated microglia critically contributes to neuroinflammation, anxiety-like behavior, and cognitive dysfunction in the mouse model of LPS-induced sepsis. Therefore, therapeutic modulation of the CD137L/CD137 signaling pathway may represent an effective way to minimize brain damage and prevent cognitive and emotional deficits associated with SAE.

## Introduction

Sepsis, defined as life-threatening multiorgan dysfunction caused by a dysregulated host response infection, is a leading cause of death in intensive care units (Singer et al., 2016). A recent report indicates a pooled incidence of 189 hospital-treated adult sepsis cases per 100,000 person-years with an estimated mortality of 26.7% (Fleischmann-Struzek et al., 2020). Sepsis-associated encephalopathy (SAE) is a central brain dysfunction caused by peripheral or systemic inflammatory infection. About 70% of patients with severe sepsis develop SAE, which increases mortality, prolongs hospitalization, and determines excessive consumption of medical resources (Helbing et al., 2018). The  symptoms of SAE range from delirium to coma (Gofton & Young, 2012). Therefore, early diagnosis and intervention are very important for the treatment of patients with sepsis. However, the pathological mechanisms of SAE have not been fully elucidated.

Microglia, the resident macrophages of the central nervous system (CNS), are closely associated with SAE pathogenesis (Li et al., 2020). Uncontrolled neuroinflammation is the main feature of SAE, and the main cause of abnormal brain function and neuronal death associated with this condition (Schwalm et al., 2014). Experiments in both humans and animals have found that microglia activation during SAE is also closely associated with emotional distress, anxiety, depression and cognitive impairment (Lemstra et al., 2007; Szollosi et al., 2018). In animal models of sepsis, microglia are typically activated by lipopolysaccharide (LPS) exposure and play a key role in mediating SAE-related behavioral changes (Osterhout et al., 2022; Cao et al., 2021). Microglia activation states can be classified into two major phenotypes, M1 and M2 (Colton, 2009; Han et al., 2018). M1 microglia produce pro-inflammatory cytokines such as IL-6, IL-1β, and TNF-α associated with tissue and nerve injury, whereas M2 microglia produce anti-inflammatory cytokines (IL-4, and IL-10), and wound-healing genes, including arginase-1 (Arg1), which promote tissue and nerve repair (Cherry et al., 2014; Jin & Yamashita, 2016). In sepsis, activated microglia eminently display an M1 phenotype that contributes to disrupting neuronal function and triggers neuronal damage by enhancing the production of inflammatory mediators (Xin et al., 2023). In turn, a recent study showed that cerebroventricular mitochondrial transplantation alleviated brain dysfunction and attenuated behavioral deficits in a mouse model of SAE by inducing microglial M2 polarization (Yan et al., 2020). Therefore, the balance between these contrasting microglial phenotypes is thought to play an essential role in the pathogenesis of SAE (Ren et al., 2020).

CD137L (TNFSF9, 4-1BBL) is a transmembrane glycoprotein that belongs to the tumor necrosis factor (TNF) ligand family and plays an important role in regulating the adaptive immune response (Watts, 2005; Croft, 2009). It is expressed on antigen-presenting cells (APCs), and its expression levels increase upon APC activation (Zeng et al., 2019). CD137L cross-links its receptor CD137 (TNFRSF9, 4-1BB), also a transmembrane glycoprotein, expressed on the surface of activated T cells, NK cells, dendritic cells (DC), and vascular endothelial cells. CD137 is a member of the TNF receptor superfamily and a potent co-stimulatory molecule in activated T cells. CD137 signaling in T cells promotes survival, enhances proliferation and effector functions, and confers metabolic sufficiency (Etxeberria et al., 2020; Tu et al., 2012). Signaling through the CD137 receptor/ligand system was shown to activate microglia, contributing to neuroinflammation and neurodegeneration in various CNS pathologies. Blocking CD137L signaling reduces the release of neuroinflammatory mediators, and CD137L knockout mice are largely prevented from developing neurodegenerative disease (Wong & Schwarz, 2020; Mak et al., 2019; Ma et al., 2013). However, the mechanism by which CD137L/CD137 signaling modulates microglia activation, and its contribution to the onset and development of SAE remain incompletely understood.

Based in the above evidence, we hypothesized that the CD137 receptor/ligand system might regulate microglia polarization to influence SAE-induced neuroinflammation and cognitive and behavioral dysfunction. Using a mouse model of sepsis and BV2 microglia cultures to assess the effects of CD137 stimulation and CD137L neutralization, in this study we highlight a critical role for the CD137/CD137L axis in promoting sepsis-induced, microglia-mediated neuroinflammation and determining anxiety-cognitive and behavioral deficits characteristic of SAE.

## Materials and Methods

### SAE Mouse Model and Treatments

Study protocols were carried out in strict accordance with the Ethical Guidelines for the Use of Animals in Research and were approved by the Institutional Animal Care and Use Committee of Guangdong Medical University (Zhanjiang, China; approval No GDY2202635). Adult C57BL/6 male mice (aged 8–10 months, 28–35 g, SiPeiFu Biotech, Beijing, China) were used to develop a SAE model. They had *ad libitum* access to an irradiated diet and were housed under a reverse light/dark cycle (12:12 h) at 23 ± 1 °C with a relative humidity of 50 ± 5%. Mice were randomly divided into 3 groups: Control (Ctrl), LPS treatment (LPS), and LPS combined with TKS-1 (LPS + TKS-1). LPS (#L2630, Sigma) was dissolved in 1 × PBS and administered (2 mg/kg) by intraperitoneal (i.p.) injection 24 h before conducting behavioral tests. To block CD137L *in vivo*, mice in the LPS + TKS-1 group were i.p. injected with the CD137L neutralizing antibody TKS-1 (200 µg; #BE0100, Bio X cell) dissolved in PBS (Nguyen et al., 2009), 2 h prior to LPS treatment. Control animals received PBS in lieu of LPS and TKS-1.

### Behavioral Tests

All behavioral test were carried out at room temperature during the light phase, and both the experimenter and the analyst were blinded to the experimental groups. The mice were handled daily for 5 days and adapted for 30 min in the behavior testing room before conducting the tests. Behavioral tests groups included the control (Ctrl; n = 7), the LPS treatment (LPS; n = 7)), and LPS combined with TKS-1 treatment (LPS + TKS-1; n = 7)). Body weight measurements were made before/after LPS-treated.

### Open Field Test (OFT)

Locomotor activity and anxiety-like behavior were assessed using the OFT. Mice were placed in the center of a white plexiglass box (50 x 50 x 50 cm) and allowed to explore freely for 5 min. The total distance traveled (a measure of locomotor activity) and time spent in the central area of the open field (a measure of anxiety-like behavior) were recorded using an overhead digital camera, and digital tracks for each mouse were analyzed by EthoVision XT software (Nodus Information Technology, Wageningen, Netherlands). The chamber was cleaned with 75% ethanol at the end of each test.

### Elevated Plus Maze (EPM) Test

Anxiety-related behavior was assessed with the EPM test. The EPM apparatus is made of an opaque cross-shaped Plexiglas structure with two intersecting planks (i.e. open and closed arms, each 77cm long and 7 cm wide) raised 70 cm above the ground. The shielding around the closed arm is 19 cm high and open at the top. The mice are placed in the same direction, facing the intersection of the maze, and allowed to freely explore the maze arms for 5 min. The time spent in the open arms was recorded to determine whether the mice showed anxiety-like behavior. Behavior detection of elevated plus maze with EthoVision XT software. The chamber was cleaned with 75% ethanol at the end of each test.

### Spontaneous Alternation Test in Y-Maze

The spontaneous alternation Y-maze test was used to assess spatial working memory in mice (Kim et al., 2014; Hsiao et al., 1995). Mice were randomly placed at the end of one arm (start arm), and allowed to explore the Y-maze for 5 min. The Y-maze consists of three identical arms, each 37 cm long, 10 cm high and 5 cm wide, interconnected at an angle of 120°. The three arms are randomly defined as A, B, and C, and spontaneous alternation occurs when a mouse enters separate arms of the maze in each of three consecutive arm entries (e.g., ABC, ACB, CAB, etc.). The percentage of spontaneous alternations was calculated as (number of spontaneous alternations)/(number of total arm entries—2) × 100% (Kim et al., 2014). Mouse behavior was recorded using an overhead digital camera, and digital tracks for each mouse were analyzed by EthoVision XT software. The chamber was cleaned with 75% ethanol at the end of each test.

### Cell Culture and Treatments

The mouse microglia cell line BV2 was obtained from Shenzhen University and cultured in high-glucose Dulbecco’s modified Eagle’s medium (DMEM) (#C11995500BT; Invitrogen/Gibco) supplemented with 2% fetal bovine serum (FBS; #AUS-015-02, Cell-Box), 1% penicillin, and 100 U/mL streptomycin (#15140-122, Invitrogen/Gibco). Cells were maintained in an incubator in a 5% CO_2_, 95% air environment at 37 °C. For all the treatments described below, BV2 cells were seeded in six-well plates at a density of 5 x 10^4^ cells/mL per well. When cell density reached 80% − 90%, the cultures were fasted in non-FBS DMEM immediately for 2 h before treatments. In the first set of experiments, the cells were randomly divided into 4 groups (n = 3–5 6-well plates/group): Control (Ctrl), TKS-1 (TKS-1 10 µg/mL; #BE0100, Bio X cell), LPS (LPS 1 µg/mL; #L2630, Sigma-Aldrich), and TKS-1 plus LPS (TKS-1 10 µg/mL + LPS 1 µg/mL). After 24 h, cells and culture media were collected for ELISA, immunofluorescence staining, flow cytometry and RNA extraction.

A second set of experiments was conducted using recombinant CD137-Fc protein (#50811-M02H, SinoBiological), to induce CD137L signaling (Yeo et al., 2012), with a human IgG1-Fc fragment (#10702-HNAH, SinoBiological) used as negative control. BV2 cells were divided into two group (n = 3–6 6-well plates/group): FC (IgG1-Fc protein, 10 µg/mL) and FC-CD137 (recombinant CD137-Fc protein, 10 µg/mL). After 1 h, cells and culture media were collected for ELISA, immunofluorescence staining and RNA extraction.

### Flow Cytometry

The effect of LPS on CD137L expression was analyzed in BV2 cells by flow cytometry. BV2 microglia cells were incubated for 24 h with anti-CD137L monoclonal antibody (TKS-1, 10 µg/mL) along with LPS (1 µg/mL). In this experiments, the cells were randomly divided into 3 groups (n = 4–5 6-well plates/group): Control (Ctrl), LPS (LPS 1 µg/mL; #L2630, Sigma-Aldrich), and TKS-1 plus LPS (TKS-1 10 µg/mL + LPS 1 µg/mL). After these treatments, the cells were collected, washed with PBS three times, and blocked in freshly prepared blocking solution (5% donkey serum and 0.5% Triton X-100 in PBS)) for 0.5 h at 4 °C. Next, the cells were incubated with primary antibodies (CD137L: Goat polyclonal antibody, R&D 1246-4L, 1:500) for 1 h. Then, the cells were washed with PBS three times and incubated for 0.5 h at 4 °C with suitable Alexa Fluor dye-conjugated IgG secondary antibodies. Flow cytometry was performed either on BD FACSAria™ II. Data analysis was performed using FlowJo software (Tree Star, Inc.).

### Enzyme Linked Immunosorbent Assay (ELISA)

Harvested BV2 cells and their supernatants, as well as hippocampal tissue, were stored at − 80 °C until processing. Expression levels of CD137L (#EK6197-2, Signalway Antibody), IL-6 (#EM0121, FineTest®), IL-1β (#PI301, Beyotime), IL-10 (#EM0100, FineTest®), and TNF-α (#EM0183, FineTest®) were measured using ELISA kits according to the manufacturer’s instructions.

### Real Time-Quantitative Polymerase Chain Reaction (RT-qPCR)

Total RNA was extracted from mouse hippocampi (n = 4–5/group) or BV2 cells (n = 3–6 6-well plates/group) using TRIzol® reagent (Invitrogen). From each sample, 500 ng of total RNA was reverse transcribed into cDNA using PrimerScript™ RT reagent kit (Takara) according to the manufacturer’s instructions. Primers were designed by Primer Premier 5.0 software. RT-qPCR was performed using SYBR Premix ExTaq kit (Takara) on an AB1 PRISM 7500 Sequence Detection System (Applied Biosystems), according to the manufacturer’s instructions. The relative expression levels of target genes were calculated using the 2^*−∆∆CT*^ method with β-actin as a normalizing gene. The following primer sequences were used for *RT*-*qPCR*:

*TLR4-F*: 5′- AGCTTCTCCAATTTTTCAGAACTTC-3′ (forward)
*TLR4-R*: 5′-TGAGAGGTGGTGTAAGCCATGC-3′ (reverse)
*IL-6-F*: 5′- TAGTCCTTCCTACCCCAATTTCC-3′ (forward)
*IL-6-R*: 5′- TTGGTCCTTAGCCACTCCTTC-3′ (reverse)
*TNF-α-F*: 5′- CTTCTCATTCCTGCTTGTGG-3′ (forward)
*TNF-α-R*: 5′- ATGAGAGGGAGGCCATTTG-3′ (reverse)
*IL-1β-F*: 5′- TTCAGGCAGGCAGTATCACTC-3′ (forward)
*IL-1β-R*: 5′- GAAGGTCCACGGGAAAGACAC-3′ (reverse)
IL-10-F: 5′- GGCAGAGAACCATGGCCCAGAA-3′ (forward)
*IL-10-R*: 5′- AATCGATGACAGCGCCTCAGCC-3′ (reverse)
*IL-4-F*: 5′- TGGGTCTCAACCCCCAGCTAGT-3′ (forward)
*IL-4-R*: 5′- TGCATGGCGTCCCTTCTCCTGT-3′ (reverse)
*YM-1-R*: 5′- ACCCCTGCCTGTGTACTCACCT-3′ (forward)
*YM-1-F*: 5′- CACTGAACGGGGCAGGTCCAAA-3′ (reverse)
*CD137L-F*: 5′- GCGGTTAATGTTCGGGATCG-3′ (forward)
*CD137L-R*: 5′- AAAGCGACTAGGCCATAGAGC-3′ (reverse)
*Aif1-F*: 5′- TGGTCCCCCAGCCAAGA-3′ (forward)
*Aif1-R*: 5′- CCCACCGTGTGACATCCA-3′ (reverse)
*β-Actin-F*: 5′- TTTGCAGCTCCTTCGTTGC-3′ (forward)
*β-Actin-R*: 5′- CCATTCCCACCATCACACC-3′ (reverse)

### Immunofluorescence Staining

For immunofluorescence staining, mice were anaesthetized by intraperitoneal injection with 1.5% sodium pentobarbital (0.09 mg/g) and intracardially perfused with saline, followed by 4% paraformaldehyde (PFA; #EL539A-1, Biosharp). The brains were then excised, post-fixed in 4% PFA for 24 hours at 4 °C, and progressively dehydrated in 10%, 20%, and 30% sucrose at 4 °C. Coronal sections of the hippocampus were cut at 25 µm on a cryostat (Leica CM1950, Germany). Sections were washed with PBS three times, and blocked in freshly prepared blocking solution (5% donkey serum and 0.5% Triton X-100 in PBS)) for 1.5 h at room temperature. Then, the sections were incubated overnight at 4 °C with primary antibodies, including anti-ionized calcium-binding adapter protein 1 (Iba1, Goat polyclonal antibody, Abcam ab5076, 1:1000; Rabbit monoclonal antibody, Abcam ab178846, 1:1000); CD68 (Rat monoclonal antibody, Abcam ab53444, 1:500); Arginase-1 (Rabbit monoclonal antibody, Cell Signaling Technology #93668, 1:500); CD137L (Goat polyclonal antibody, R&D 1246-4L, 1:500); TNF-α (Rabbit monoclonal antibody, Cell Signaling Technology #11948, 1:500); and IL-6 (Rabbit polyclonal antibody, Affinity Biosciences #DF6087, 1:500). The next day, the brain slices were washed with PBS three times and incubated for 2 h at room temperature with suitable Alexa Fluor dye-conjugated IgG secondary antibodies (ThermoFisher Scientific A-11055, A-21202, A-31570, A78945, A-31572, A-21432, 1:500). After being washed with PBS, sections were counterstained with 4,6-diamidino-2-phenylindole (DAPI; #C1005, Beyotime), mounted with Vectashield Antifade mounting medium (#H1000-10, Vector), imaged by confocal microscopy (LSM 800, Carl Zeiss), and processed for quantitative analysis using ImageJ software.

For cytoimmunofluorescence, BV2 cells were fixed with 4% PFA for 15 min at room temperature, washed with PBS, and subjected to the immunolabeling steps described above.

### Statistical Analyses

All data were analyzed with GraphPad Prism 7 software using either unpaired two-tailed *t* tests with Welch’s correction, for comparisons between two groups, or one-way ANOVA, for multiple comparisons. Data are presented as the mean ± SEM. ANOVA followed by Bonferroni post hoc test. *p < 0.05 was considered significant.

## Results

### LPS—Induce Sepsis Upregulates CD137L Expression in Activated Microglia

**Fig. 1:**
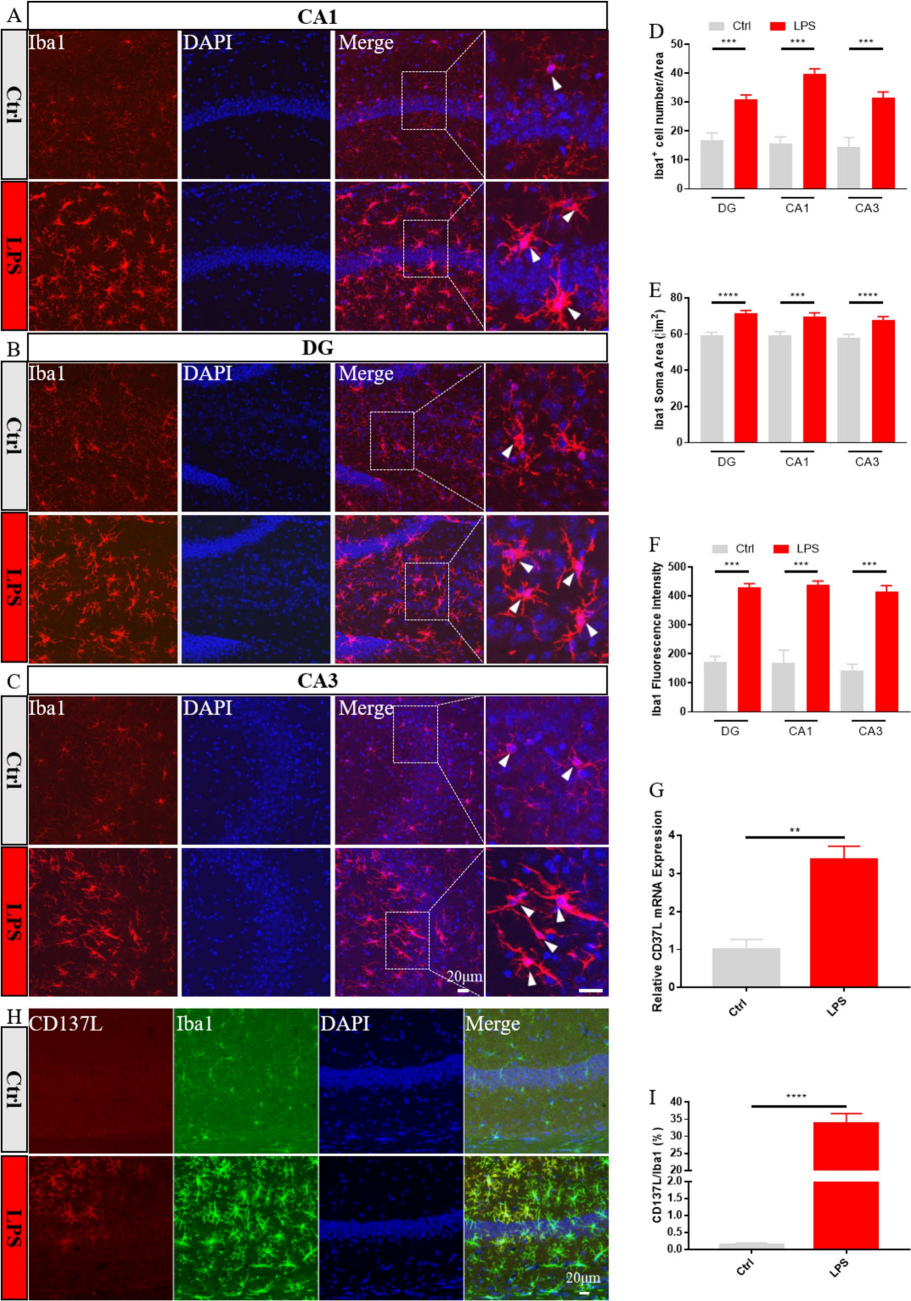
CD137L expression is increased in activated hippocampal microglia of mice with LPS-induced SAE. **A–C** Representative immunofluorescence images of Iba1-labeled microglia in the mouse hippocampus. **D** Quantification of mean Iba1-positive cell numbers/area from 10x field (Ctrl group, n = 8–17 from 5 mice; LPS group, n = 12–17 from 6 mice). **E** Quantification of mean Iba1-positive somatic area (Ctrl group, n = 30 from 3 mice; LPS group, n = 30 from 3 mice). **F** Quantification of the fluorescence intensity of Iba1-positive cells in the DG, CA1, and CA3 regions (n = 3–4 mice/group). **G** Analysis of hippocampal CD137L mRNA levels by RT-qPCR (Ctrl group n = 4; LPS group n = 6). **H** Representative dual-immunofluorescence images of Iba1 (green) and CD137L (red) co-localization in the hippocampus. **I** Quantification of the proportion of CD137L-positive microglia (n = 3–4 mice/group). Data are shown as mean ± SEM; ***p ≤ 0.001, ****p ≤ 0.0001

Immunofluorescence detection of Iba1, a pan-microglial marker whose expression increases with microglial activation, was first used to evaluate microglia activation in the hippocampus of control and SAE model mice at 24 h post-treatment. Results showed that Iba1 expression was markedly increased in the DG, CA1, and CA3 regions of LPS-treated, compared to control, mice (Fig. 1A–C, F). Meanwhile, parallel quantification of Iba1 fluorescence showed that LPS exposure significantly enhanced the number and somatic area of hippocampal microglia, compared to the control group (Fig. 1D, E). To assess whether CD137L is expressed in microglia during LPS-induced sepsis, CD137L expression was detected in the mouse hippocampus using RT-qPCR and immunofluorescence assays. Compared with the control group, increased CD137L mRNA levels were observed in the LPS-treated group (Fig. 1G). Similarly, immunofluorescence staining results showed that CD137L protein levels in the hippocampus were concomitantly increased after LPS exposure (Fig. 1H, I). In addition, the increased CD137L was all co-labeled with microglia, whereas not all activated microglia also expressed CD137L.

**Fig. 2:**
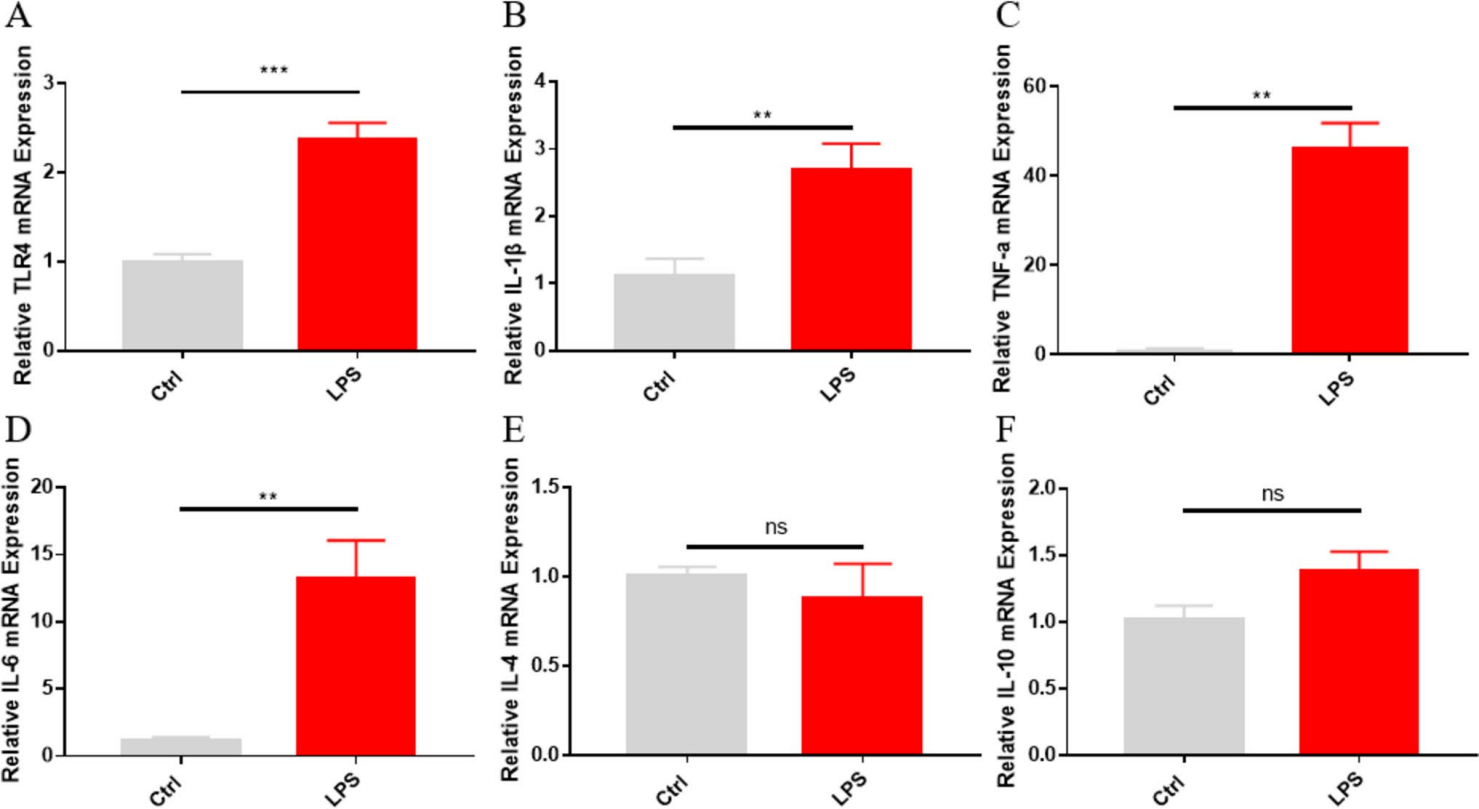
Analysis of microglia-associated, inflammation-related cytokine expression in the hippocampus of LPS-treated mice. **A–D** RT-qPCR data for M1 markers: **A** TLR4 (Ctrl group, n = 4 mice; LPS group, n = 5 mice); **B** IL-1β; **C** TNF-α; **D** IL-6 (B-D, n = 5 mice/group). **E, F** RT-qPCR data for M2 markers (n = 5 mice/group): **E** IL-10; **F** IL-4. Data are shown as mean ± SEM; **p ≤ 0.01, ***p ≤ 0.001

**Fig. 3:**
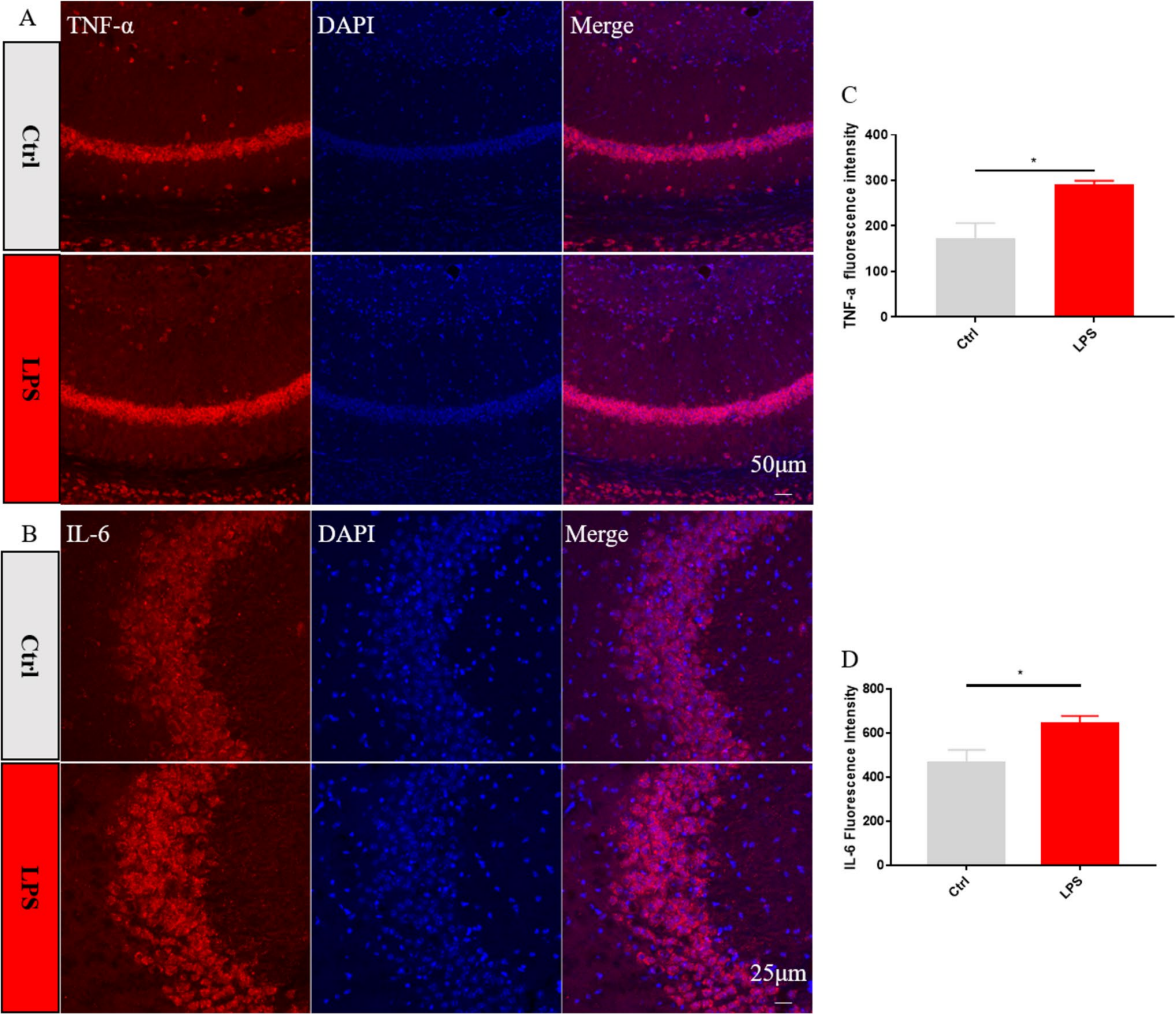
Immunofluorescence analysis of TNF-α and IL-6 expression in hippocampal microglia. **A** Representative images of TNF-αimmunofluorescence. **B** Representative images of IL-6 immunofluorescence. **C** Quantification of mean TNF-α fluorescence intensity (n = 4 mice/group). **D** Quantification of mean IL-6 fluorescence intensity (n = 4 mice/group). Data are shown as mean ± SEM; ***p ≤ 0.001, ****p ≤ 0.0001

Toll-like receptor (TLR4), which is expressed on the surface of microglia, is characteristically activated by LPS (Zusso et al., 2019). Interestingly, LPS was shown to induce TLR4-CD137L binding to mediate pro-inflammatory gene expression in macrophages (Kang et al., 2007). RT-qPCR assay results showed that compared with control animals, mRNA levels of TLR4 (Fig. 2A) and pro-inflammatory cytokines (IL-6, IL-1β, and TNF-α) (Fig. 2B–D) were significantly increased in the hippocampus of LPS-treated mice. In contrast, there was no significant difference in the mRNA expression of the anti-inflammatory factors IL-10 and IL-4 between LPS-treated and control mice (Fig. 2E, F). We also detected protein levels of pro-inflammatory cytokines (IL-6 and TNF-α) using immunofluorescence staining. Results showed that IL-6 and TNF-α immunoreactivity was significantly increased in LPS-treated mice (Fig. 3).

**Fig. 4:**
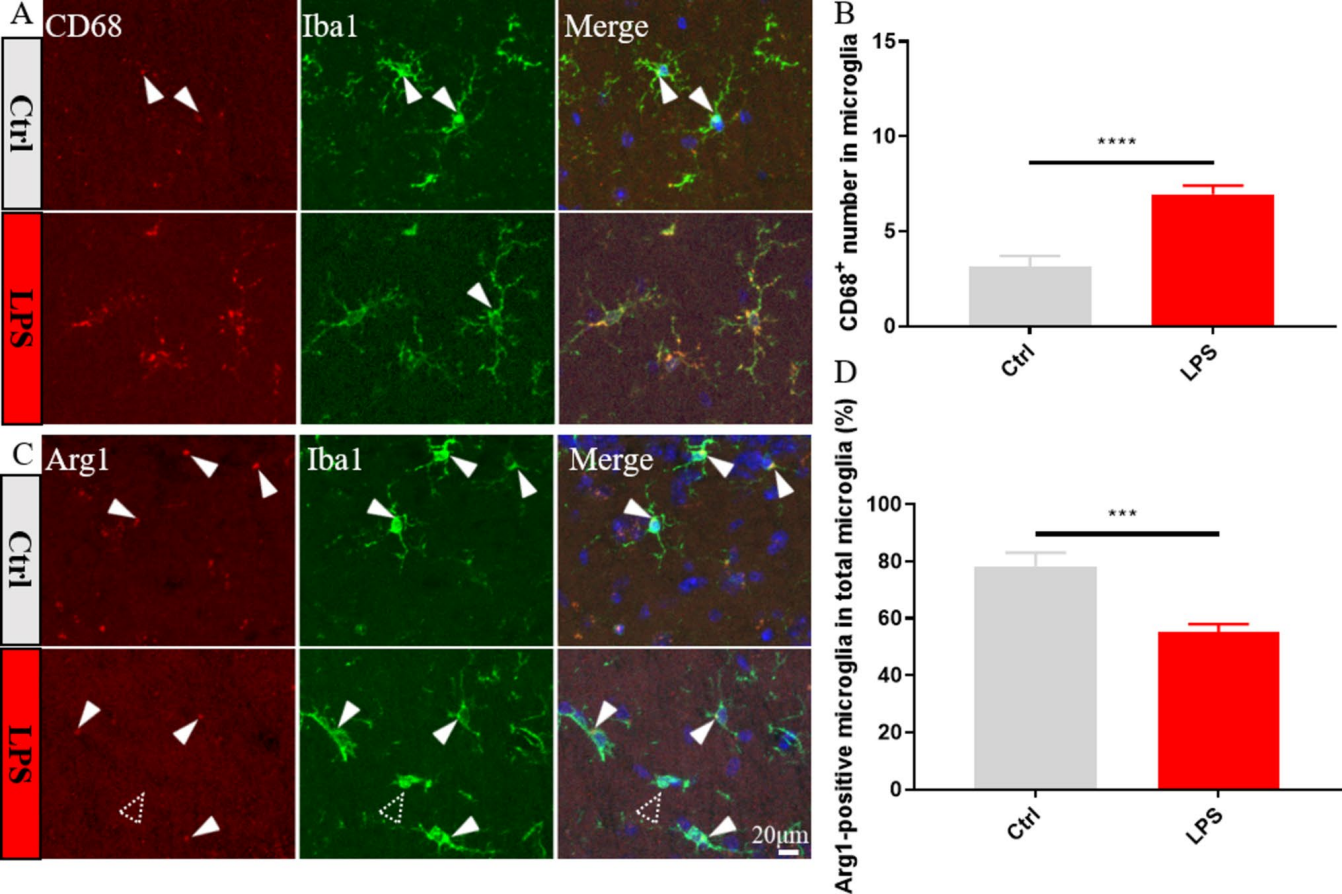
Analysis of phenotypic markers of M1 and M2 polarization in hippocampal microglia. **A** Representative images of hippocampal slices co-labeled with antibodies against Iba1 (green) and the M1 marker CD68 (red). **B** Quantification of the number of CD68/Iba1-positive microglia from 20x field (n = 26–50 cell from 3 mice/group). **C** Representative images of hippocampal slices co-labeled with antibodies against Iba1 (green) and arginase 1 (Arg1, red). **D** Quantification of mean Arg1 fluorescence intensity (n = 25–26 slice from 3 mice/group). Solid arrowheads indicate positive CD68 and Arg1 labeling, and hollow arrowhead denotes negative Arg1. Data are shown as mean ± SEM; *p ≤ 0.05; **p ≤ 0.01

Next, we measured by immunofluorescence the effect of LPS exposure on phenotypic markers of M1 (CD68) and M2 (Arg1) microglia. Results showed that the expression of CD68 was significantly increased (Fig. 4A, B), while that of Arg1 was significantly decreased (Fig. 4C, D), in LPS-treated mice. These results suggested that SAE- mediated microglia activation is paralleled by upregulation of CD137L in these cells.

### CD137 Stimulation Enhances CD137L Expression in Microglia to Trigger Neuroinflammation

**Fig. 5:**
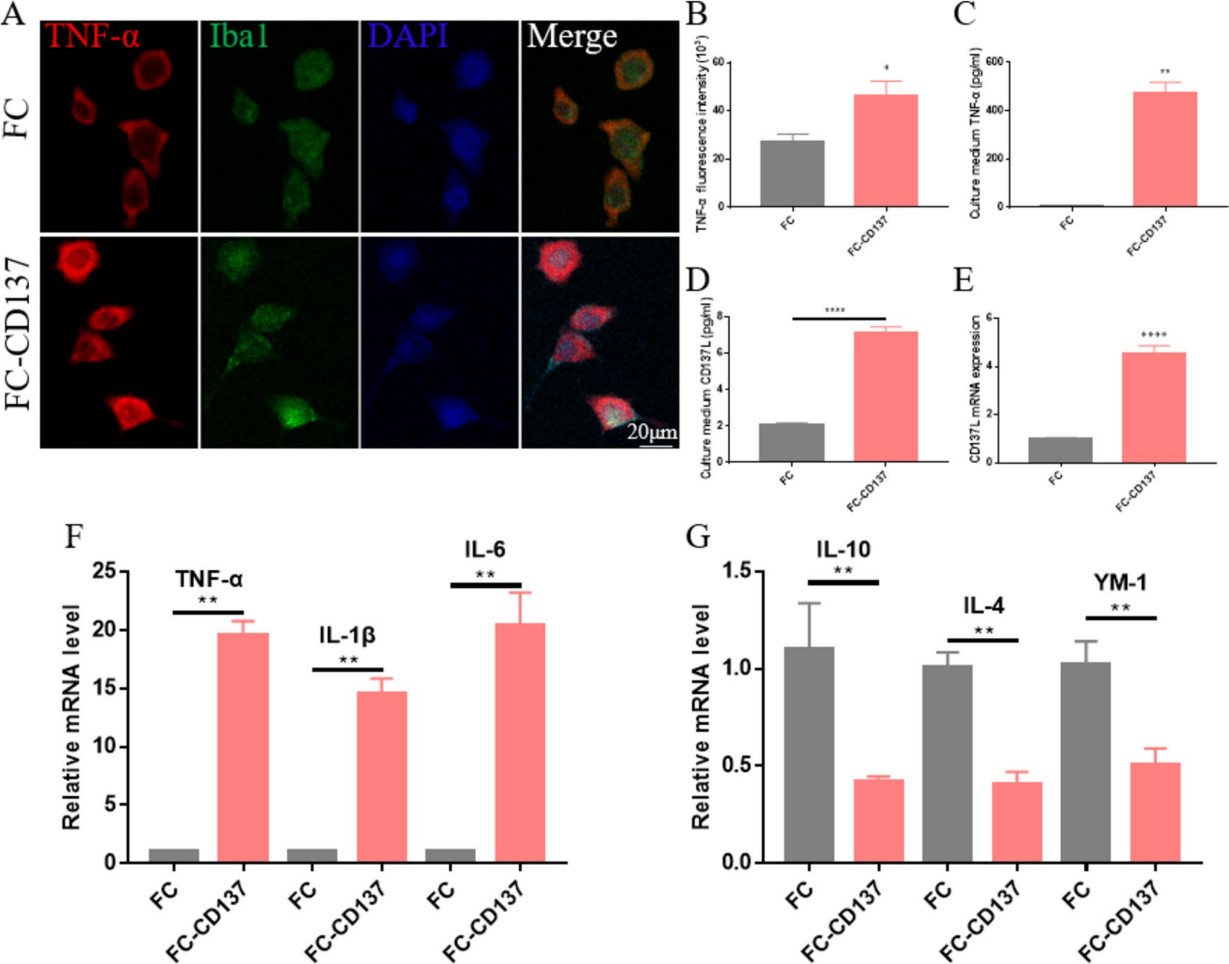
CD137 stimulation induces M1 polarization in microglia in vitro. **A** Representative images of TNF-α and Iba1 dual-immunofluorescence in cultured BV2 microglia alternatively treated with IgG1-Fc isotype control (FC) or recombinant CD137-Fc protein (FC-CD137). **B** Quantification of TNF-α mean fluorescence intensity (n = 11/group). **C, D** ELISA-based analysis of TNF-α (C) and CD137L (D) levels in culture media from BV2 cells. (n = 3–6/group). **E** Detection of CD137L mRNA levels in BV2 microglia by RT-qPCR (n = 5/group). **F** Detection of M1 phenotype markers (TNF-α, IL-1β, and IL-6) in cultured BV2 microglia by RT-qPCR (n = 6/group). **G** Detection of M2 phenotype markers (IL-10, IL-4, and YM-1) in cultured BV2 microglia by RT-qPCR (n = 3–6/group). Data are shown as mean ± SEM; *p ≤ 0.05; **p ≤ 0.01; ****p ≤ 0.0001

Subsequently, we investigated whether activation of CD137L/CD137 signaling regulates microglia-derived pro-inflammatory cytokine release. It has been reported that recombinant CD137-Fc fusion protein (FC-CD137) treatment induces the expression of CD137L in neural stem cells (Yun et al., 2013; Yeo et al., 2012). Thus, we studied the effect of CD137-Fc on CD137L expression and its relationship with microglia-related inflammatory responses in cultured mouse BV2 microglial cells. Consistent with activation of CD137L/CD137 signaling, immunofluorescence staining and ELISA results showed that the expression of TNF-α was significantly enhanced by FC-CD137 exposure (Fig. 5A–C). RT-qPCR and ELISA results showed that compared with the IgG1-Fc isotype (FC) control group, after 1 h treatment both mRNA and protein levels of CD137L were significantly increased in FC-CD137-treated cells (Fig. 5D, E). Similarly, RT-qPCR assays revealed that in FC-CD137-treated BV2 microglia mRNA levels of pro-inflammatory cytokines (IL-6, IL-1β, and TNF-α) were significantly increased, while those of anti-inflammatory cytokines (IL-10, IL-4, and YM-1) were significantly decreased, compared with the FC control group (Fig. 5F, G). These findings suggest that CD137 overexpression in microglia upregulates the expression of CD137L to promote a pro-inflammatory response.

### Antibody-Based Inhibition of CD137L Reduces Pro-inflammatory Cytokine Release in BV2 Microglia In Vitro

**Fig. 6:**
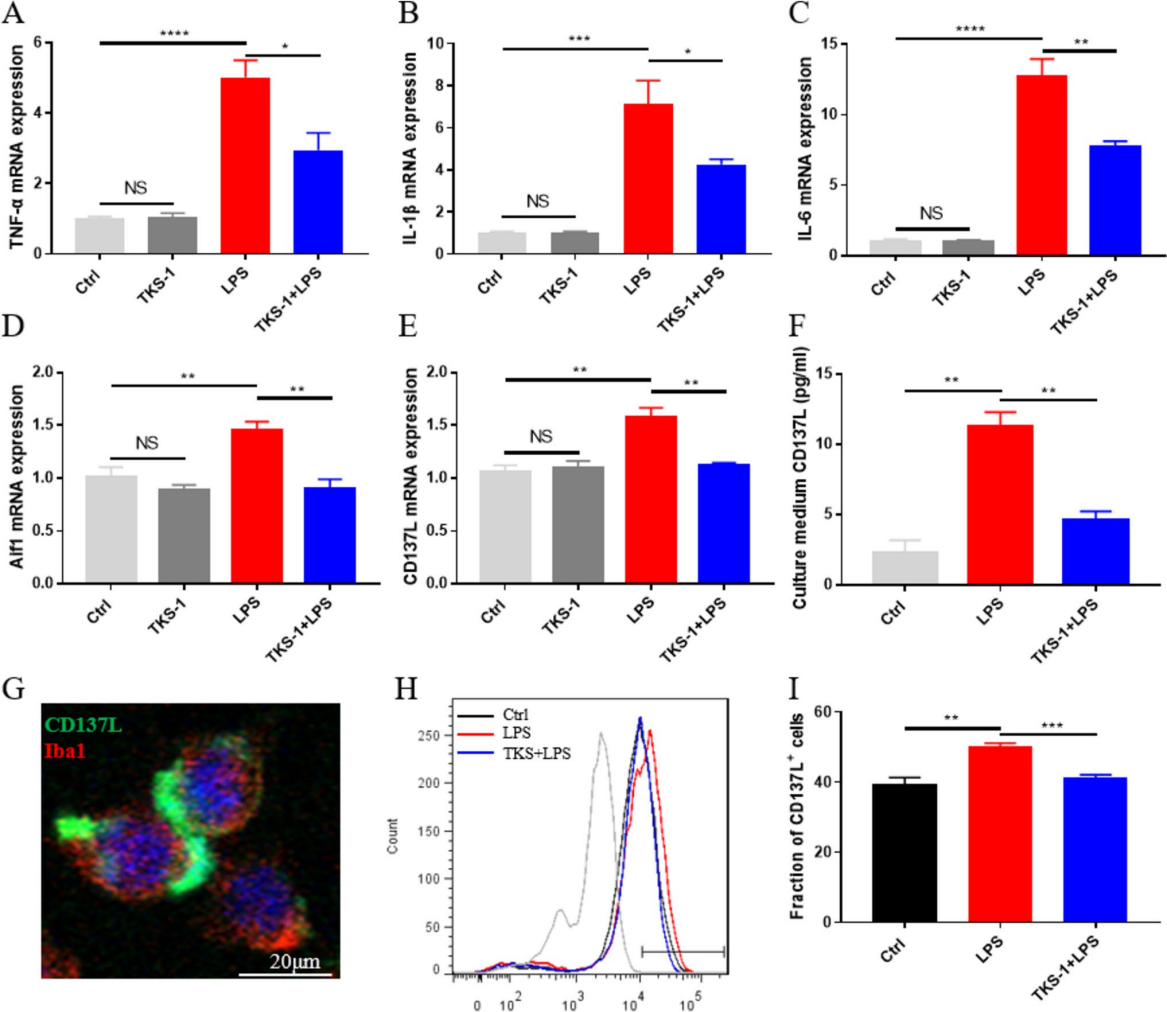
TKS-1-mediated CD137L inhibition attenuates activation of microglia in vitro. **A–E** RT-qPCR data for **A** TNF-α, **B** IL-1β, **C** IL-6, **D** Aif1, and **E** CD137L expression (n = 4–5/group) in the control, TKS-1, LPS, and TKS-1 + LPS cell groups. **F** ELISA-based measurements of CD137L concentration in culture media from BV2 cells (n = 3–4/group). **G** Representative dual-immunofluorescence image showing colocalization of CD137L and Iba1 in BV2 cells. **H**,** I** Representative flow cytometry histograms **H** and corresponding quantification **I** of CD137L expression in cultured BV2 microglia cells (n = 4–5/group). Grey histogram: Isotype control. Black histogram: Control group. Red histogram: LPS group. Blue histogram: TKS-1 group (anti-CD137L monoclonal antibody). Numbers in panels indicate the percentage of CD137L^+^ cells. Data are shown as mean ± SEM; *p ≤ 0.05; **p ≤ 0.01; ****p ≤ 0.0001

To validate the involvement of the CD13L/CD137 axis in the pro-inflammatory microglial response identified above, we treated BV2 cells with TKS-1, a CD137L neutralizing antibody that was shown to reduce the expression of CD137L both in vitro and in vivo (Mbanwi et al., 2017; Wakley et al., 2018). In line with our findings in the mouse hippocampus, RT-qPCR analysis showed that IL-6, IL-1β, and TNF-α mRNA expression was significantly increased in LPS-treated BV2 cells. Suggesting a pivotal role for CD137L/CD137 signaling in pro-inflammatory cytokine expression in activated microglia, these changes were significantly reversed in LPS-activated, TKS-1-treated BV2 microglia (Fig. 6A–C). Confirming that LPS exposure effectively activated microglia in vitro, increased mRNA expression of Aif1 (a microglial activation marker) was detected in LPS-treated BV2 cells. In turn, indicating a stimulatory effect of CD137L expression on microglial activation, TKS-1 treatment reduced the expression of Aif1 mRNA in the presence of LPS (Fig. 6D).

RT-qPCR, ELISA, and flow cytometry analysis results showed that CD137L expression was significantly enhanced at both the mRNA and protein levels in LPS-stimulated BV2 microglia in comparison with control cells. In turn, LPS-mediated CD137L expression was abrogated in cells co-treated with the TKS-1 (Fig. 6E–I). These results indicated that TKS-1-mediated suppression of CD137L mRNA and protein expression in LPS-activated BV2 microglia inhibits the transcription of pro-inflammatory cytokines.

### In Vivo TKS-1-Mediated Inhibition of CD137L Reduces Microglia-Derived Pro-inflammatory Cytokine Production

**Fig. 7:**
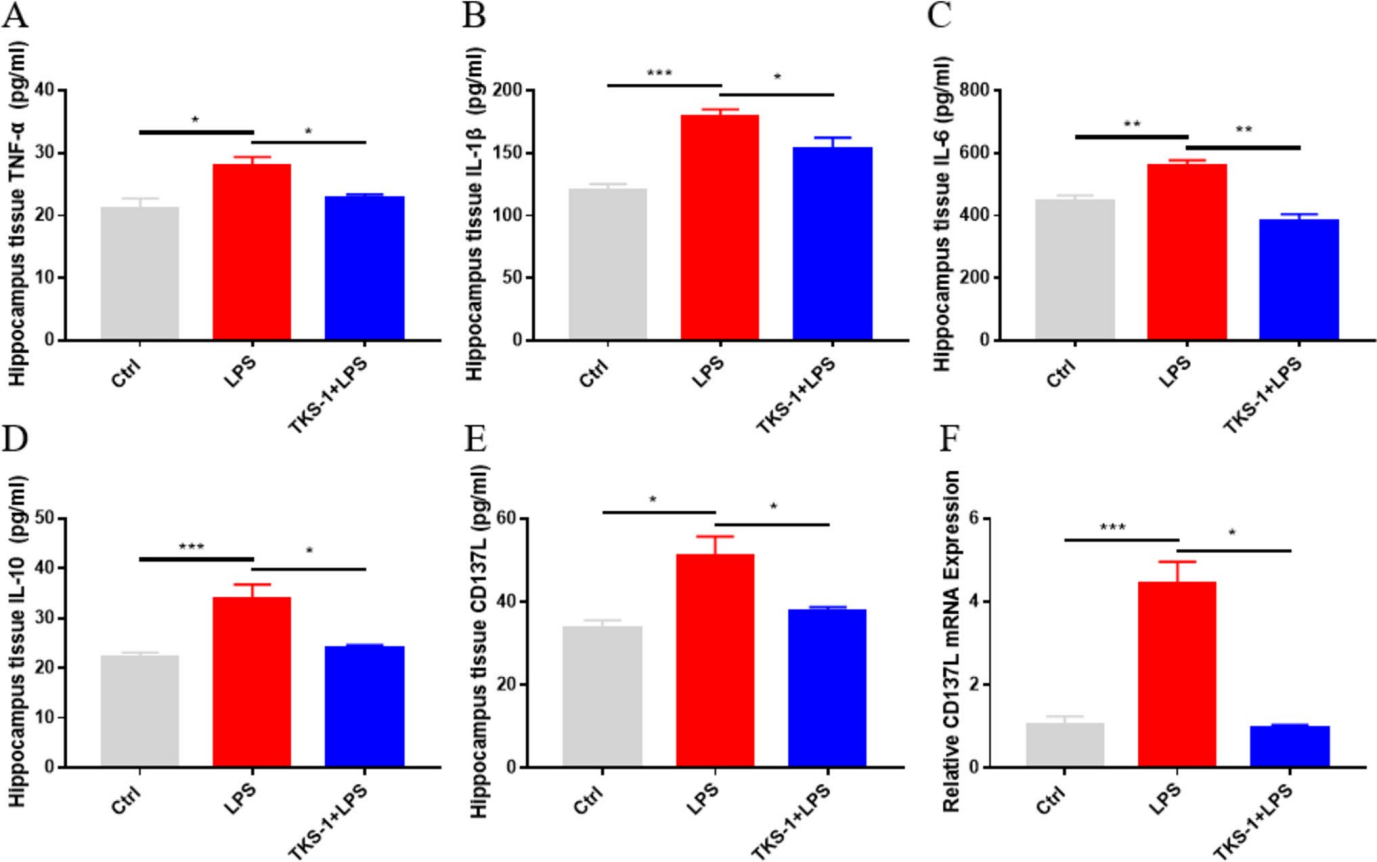
TKS-1 inhibits hippocampal CD137L expression and pro-inflammatory cytokine production in mice with LPS-induced SAE. **A–E.** ELISA-based analysis of **A** TNF-α, **B** IL-1β, **C** IL-6, **D** IL-10, and **E** CD137L levels in hippocampi from control, LPS, and TKS-1 + LPS mice. **F** Detection of hippocampal CD137L mRNA levels by RT-qPCR. n = 4 mice/group. Data are shown as mean ± SEM; *p ≤ 0.05; **p ≤ 0.01; ***p ≤ 0.001; ****p ≤ 0.0001

To evaluate if the in vitro effects of the CD137L neutralizing antibody TKS-1 are reproduced in vivo, TKS-1 was intraperitoneally injected to mice before LPS administration. ELISA assays showed that compared with the control group, hippocampal TNF-α, IL-1β, IL-6, and IL-10 levels were increased after treatment with LPS, and these changes were attenuated or prevented by TKS-1 (Fig. 7A–D). Meanwhile, RT-qPCR and ELISA results further indicated that CD137L expression was significantly enhanced in hippocampi from LPS-treated mice, and normalized instead to near control levels in mice pre-treated with TKS-1 (Fig. 7E, F). These findings suggested that TKS-1 reduces the expression of CD137L and ameliorates neuroinflammation in mice with LPS-induced SAE.

### TKS-1 Reduces Anxiety-Like Behavior and Improves Spatial Memory in Mice with LPS-Induced SAE

**Fig. 8:**
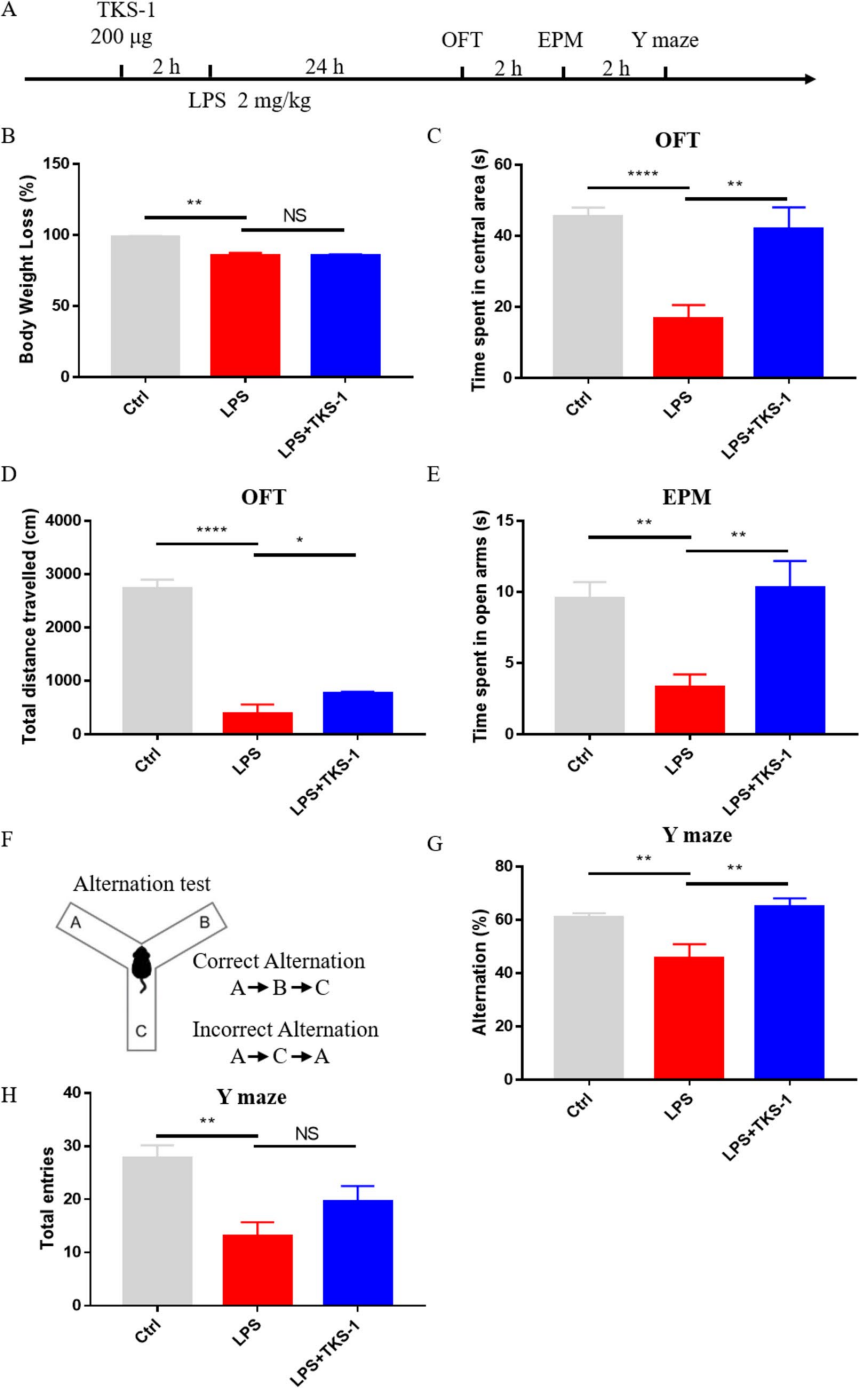
TKS-1 reverses behavioral and cognitive deficits in mice with LPS-induced SAE. **A** Time schedule of the experimental protocol. **B** Body weight measurements, taken 24 h after experimental treatments (Ctrl group, n = 8; LPS group, n = 11; TKS-1 + LPS group, n = 11). **C** Average time spent in the central area in the OFT (n = 7 mice/group). **D** Total travel distances in the OFT (n = 7 mice/group). **E** Average time spent in the open arms in the EPM test (n = 7 mice/group). **F** Schematic of Y-maze spontaneous alternation test for assessing working memory ability. **G** Percentage of alternation in Y maze (n = 11 mice/group). **H** Quantification of total arm entries in the Y maze task (n = 11 mice/group). Data are shown as mean ± SEM; *p ≤ 0.05; **p ≤ 0.01; ***p ≤ 0.001

We finally assessed whether CD137L inhibition alleviates behavioral deficits in SAE mice. Behavioral tests, including open field test (OFT), elevated plus maze (EPM) test, and Y-maze spontaneous alternation test, were conducted 24 h after treatments in the Ctrl, LPS, and LPS + TKS-1 groups of mice (Fig. 8A). At this time point, body weight measurements showed that relative to control mice, comparable and significant weight loss occurred in LPS-challenged mice treated or not with TKS-1 (Fig. 8B). OFT results demonstrated increased anxiety-like behavior, as well as reduced locomotor activity (indicated respectively by a lower total time spent in the central area of the field and decreased total distance traveled) in LPS-treated compared to control mice. Notably, these behavioral deficits were partially mitigated in LPS + TKS-1 mice (Fig. 8C, D). EPM results confirmed in turn increased anxiety-like behavior, indicated by a lower total time spent in the open arms of the elevated maze, in LPS-treated compared to control mice, and reversal of this behavior in LPS + TKS-1 mice (Fig. 8E). Lastly, reduced spontaneous alternation behavior, indicative of impaired spatial working memory, was noted in LPS-treated compared to control mice in the Y-maze test. This spontaneous alternation deficit was corrected by administration of TKS-1 (Fig. 8F, G). However, the total number of arm entries was not significantly restored in LPS + TKS-1 group mice (Fig. 8H). These findings suggest an essential role for CD137L in behavioral manifestations of SAE in mice.

## Discussion

Sepsis is a serious medical condition and a common cause of death in intensive care unit patients. Worryingly, most survivors of severe sepsis show cognitive impairment and psychological problems (Bruck et al., 2018; Calsavara et al., 2018). Animal sepsis models (commonly established by LPS injection, cecal slurry injection, or cecal ligation and puncture (CLP)) show that systemic inflammation leads to acute brain dysfunction, also known as SAE (Li et al., 2022; Manabe & Heneka, 2022). Therefore, there is an urgent need to understand how systemic inflammation leads to CNS dysfunction to help develop effective strategies for the treatment of SAE. Here, we show that inhibition of CD137L signaling remarkably attenuates neuroinflammation and cognitive and behavioral deficits in mice following peripheral LPS challenge.

CD137L acts as an acute pro-inflammatory mediator in the CNS. Similar to previous studies (Yeo et al., 2012), we showed that CD137L is significantly induced in the CNS after LPS injection. In turn, administration of TKS-1, a CD137L neutralizing antibody, prior to LPS treatment markedly reduced hippocampal IL-6, IL-1β, and TNF-α levels. Although we cannot rule out the presence of residual CD137L signaling activity in these experiments, the decrease in hippocampal transcriptional and translational levels of CD137L following TKS-1 application suggests effective inhibition of CD137L activity in the CNS. In future research, conditional knockout of CD137L in mice may help solve this concern. Similar to our CNS findings, activation of CD137L signaling was shown to induce pro-inflammatory cytokine expression in peripheral macrophages in vitro and in vivo and promote macrophage apoptosis in vitro (Kim et al., 2011; Stoll et al., 2021; Xu et al., 2021; Wei et al., 2022). Taken together, these data demonstrate that CD137L acts as an important pro-inflammatory mediator in the CNS during LPS-induced systemic inflammation.

Studies in septic animals and patients have described changes in microglia polarization status (Xin et al., 2023). Bidirectional transition between the M1 (pro-inflammatory) and the M2 (anti-inflammatory) phenotypes in microglia occur in response to different physiopathological environments. Thus, microglia polarization is considered an important target for treatment of SAE. M1 microglia was shown to contribute to SAE and sepsis-associated chronic pain (Li et al., 2020). In contrast, the role of M2-polarized microglia in SAE remains unclear. In line with previous studies (Wakley et al., 2018), we found that activation of CD137L signaling induced M1 and reduced M2 polarization in a murine model of SAE. Since, ligation of CD137L with TKS-1 inhibited M1 polarization, targeting CD137L may protect the brain from neuroinflammation induced by activated microglia by reducing M1conversion.

Studies have shown that sepsis may lead to long-term neurobehavioral changes in both people and animal models (Semmler et al., 2013; Anderson et al., 2015). Our study confirmed that mice subjected to LPS-induced sepsis exhibit both increased anxiety-like behavior and cognitive impairment. Notably, we showed that TKS-1 administration significantly alleviated SAE-related neurobehavioral deficits. This suggests that the CD137L/CD137 axis may critically influence cognitive and behavioral dysfunction in septic patients, possibly in relation with its central role in neuroinflammation.

Our work has some limitations. First, we detected LPS-induced changes in inflammatory mediators and CD137L expression only in the hippocampus. Since, other brain regions critical for cognition and behavior, such as the prefrontal cortex and the amygdala, are importantly affected by sepsis, the changes referred above need to also be investigated in these regions. Second, in this study TKS-1 was applied by intraperitoneal injection, and hence CD137L blockade was not brain-specific. This concern may be adequately addressed by directly injecting TKS-1 or CD137L-targeting vectors into the CNS. Third, we used only male mice a single strain. Gender affects behavioral output, and the role of female mice in LPS-induced behavioral impairment should be explored in the future. Lastly, the mechanism by which CD137L mediates microglia polarization was not specifically addressed in this work, and thus needs to be clarified in future research.

In summary, this is, to our knowledge, the first study investigating the contribution of the CD137L signaling system to SAE development. Our results suggest a fundamental role for CD137L in microglia activation, neuroinflammation, and neurobehavioral deficits triggered by acute sepsis. Specifically, we found that treatment with TKS-1 reduced the synthesis and release of pro-inflammatory cytokines, decreased anxiety-like behavior, and improved spatial memory in mice with LPS-induced SAE. These findings suggest that targeting the CD137L signaling pathway in microglia may represent a promising way to treat SAE.

## Data Availability

All data generated or analyzed during this study are included in this article. Further inquiries can be directed to the corresponding author.
